# High-Temperature Strain Gauge Measurement Techniques for Temperatures Above 800 °C: A Review

**DOI:** 10.3390/ma18071588

**Published:** 2025-04-01

**Authors:** Wenrui Wang, Rui Zong, Dongyue Li, Jiaming Zhang, Guangrong Teng, Shengxiang Li

**Affiliations:** 1 School of Mechanical Engineering, University of Science and Technology Beijing, Beijing 100083, China; zongqiongyu@163.com (R.Z.); lidongyue@ustb.edu.cn (D.L.); zhangjm@ustb.edu.cn (J.Z.); 2 Sichuan Gas Turbine Research Institute, China National Aviation Development Corporation, Mianyang 610500, China; tenggr2025@163.com (G.T.); lsx19920713@163.com (S.L.)

**Keywords:** high-temperature strain, contact strain measurement, sensitive grids, protective layer, transition layer

## Abstract

Pre-study tests and strain measurements of critical structural components in high-temperature environments are paramount. In the field of high-temperature strain measurement, the contact strain measurement method has the widest application range, a high measurement accuracy, and a low cost. At present, a variety of high-temperature strain gauges have been developed at home and abroad. However, due to material and processing process limitations, the research on high-temperature strain gauges applied above 800 °C is still relatively small and immature. Therefore, it is very necessary to do a systematic analysis of the research in this field. This paper describes the basic principle and structure of high-temperature strain gauges and systematically analyses and summarizes the current research status of high-temperature strain gauges’ sensitive grids, protective layers, and transition layers in terms of materials and structures. Finally, based on the existing research, it provides ideas and prospects for future contact strain measurement methods applied to higher temperatures.

## 1. Introduction

Strain gauges are sensors used to sense the strain of a component under test and can be mounted directly on the surface of the component under test. According to the operating temperature region, the strain gauges applied above 500 °C are generally called high-temperature strain gauges [1]. According to the form of the sensitive grid material, strain gauges can be classified into four types: filament type, foil type, thin-film type, and thick-film type. Foil-type strain gauges are produced by etching and other processes and are difficult to use for strain measurements above 500 °C. Thick-film strain gauges generally have a large temperature coefficient of resistance (TCR) and are also difficult to use for high-temperature strain measurements.

**Figure 1:**
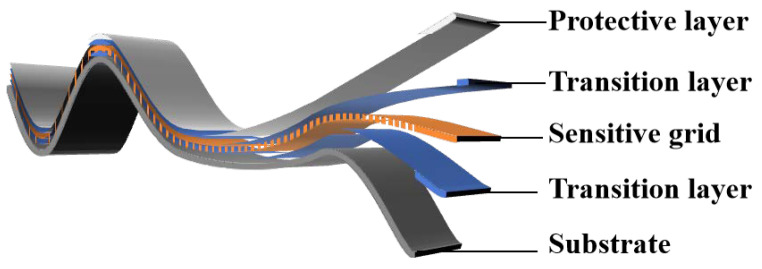
Structure diagram of high-temperature strain gauge.

At present, in the field of high-temperature strain measurement, the main research still focuses on the field of filament strain gauges and thin-film strain gauges. Both types of high-temperature strain gauges are mainly composed of the protective layer, sensitive grids, transition layer, and substrate; the structure schematic diagram is shown in Figure 1. The major difference between the two types of high-temperature strain gauges lies in the production method of the sensitive grid; the wire type high-temperature strain gauge sensitive grid is made by directly winding a metal resistor wire with a diameter of 0.015–0.03 mm, and the thin-film type high-temperature strain gauges sensitive grid is generated directly on the substrate using vacuum deposition or sputtering methods [2].

As shown in Figure 1, the protection layer is mainly used to protect the sensitive grids, which need to have better high-temperature oxidation resistance and electrical insulation. The transition layer is mainly used to bond the sensitive grids on the substrate, which needs to have good strain transfer capability, bonding strength, and electrical insulation. As the core element of high-temperature strain gauges, the sensitive grid is used to sense the strain of the measured component and convert the strain signal into a resistance signal, which needs to have a stable and high resistivity, a stable and close to 0 temperature coefficient of resistance (TCR), a high strain sensitivity coefficient, and a high working temperature. The substrate is mainly used for fixing sensitive grids and needs to have good adhesive properties, mechanical strength, and electrical insulation [3].

High-temperature strain gauges are mainly used for strain measurement of structures in high-temperature environments. Specifically, for engineering applications, they are mainly used for testing and evaluation of thermal protection structures, which can visually and effectively understand the durability and life of components, carry out timely maintenance and updating, improve the safety of the system, and avoid the occurrence of disasters. At the same time, the high-temperature strain gauges can also be in the pre-study stage of the component test, in advance to obtain the strength of the component information, optimize the design, and upgrade, to avoid a lot of strength problems in the model development [4].

With the emergence and upgrading of high-temperature-resistant materials, the rapid development of aerospace and nuclear power technology, and other cutting-edge fields, the operating temperature of core components continues to rise and breakthroughs. For example, to achieve aero-engines with higher Mach numbers and higher thrust-to-weight ratios, the turbine inlet temperature needs to be constantly raised, and the blade temperatures at all levels will also be raised. To achieve accurate measurements of strain in key components, it is necessary to improve the permissible temperature of high-temperature strain gauges. Currently, most of the more mature high-temperature strain gauges have a permissible temperature below 800 °C, and there are relatively few studies on high-temperature strain gauges applied to temperatures above 800 °C [5].

This paper systematically reviews the technology of high-temperature strain gauges used at temperatures above 800 °C. It summarizes the measurement principle of these gauges and analyzes their breakthroughs, innovations, and shortcomings. Based on the current research, this paper also explores the future development direction of strain gauges for higher temperatures.

## 2. Application Fields

In aerospace, core components often operate in extremely high-temperature environments, especially aero-engines and gas turbines. These components are exposed to high temperatures, high pressures, and severe vibration conditions for long periods of time, and structures such as blades are prone to fatigue damage and failure. By installing high-temperature strain gauges on these critical structures, strain data can be monitored in real-time. Inspectors assess the health of the components based on these data so that potential safety hazards can be detected and repaired in a timely manner. This technology effectively improves the reliability and extends the service life of aerospace vehicles [6,7].

**Figure 2:**
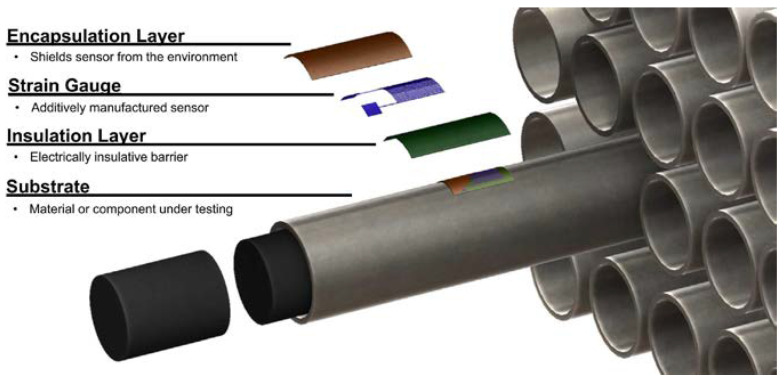
Schematic of an additively manufactured strain gauge on a nuclear fuel cladding [9].

In the field of nuclear energy, it is crucial to ensure the safe operation of reactors and their related equipment. High-temperature strain gauges, which can work stably in high-temperature environments and strong radiation fields, have successfully solved the challenge of in situ strain monitoring of core components such as the pressure vessel of a nuclear reactor and the cluster of heat transfer tubes of a steam generator, as shown in Figure 2. With their micron-level deformation capture capability, these strain gauges can quantitatively characterize the creep properties of nuclear-grade equipment materials and the expansion trend of stress corrosion cracks so that potential anomalies can be detected in a timely manner and necessary repairs and maintenance can be carried out, thus guaranteeing the stability and safety of nuclear energy facilities [8,9].

In the automotive field, the in-depth application of high-temperature strain gauges has become a key support for improving heat engine efficiency and environmental performance. High-temperature strain gauges can be used in high-temperature operating environments to help monitor, in real-time, the stress distribution and operating status of high-temperature components, such as engine combustion chamber blocks, turbochargers, and three-way catalytic devices, to optimize combustion efficiency and improve environmental performance. Further applications include the use of strain gauge technology to diagnose turbo lag in advance and extend the life of the supercharger system. The technology not only improves the energy efficiency and reliability of automotive powertrains but also provides data support for the development of new high-temperature materials through the construction of strain and temperature databases, accelerating the transition of the automotive industry towards high efficiency and low carbon use [10,11].

In the petrochemical and energy sectors, high-temperature strain gauge technology is driving the petrochemical and energy equipment monitoring paradigm toward intelligence. For key equipment, such as catalytic cracking reactors, supercritical boiler couplings, and energy transmission pipelines, high-temperature strain gauges have been deployed to realize high-precision strain measurements in high-temperature environments. This, in turn, effectively solves the problem of reconstructing the strain field on the surface of the equipment under high-temperature and high-pressure environments, and can accurately analyze the evolution of thermo-mechanical fatigue damage. It can give early warning of creep damage and improve the sensitivity of crack detection. It provides important support for accurately predicting the life of the equipment and improving the safety of the equipment [12,13,14].

## 3. Measurement Basics

The principle of high-temperature strain gauge measurement is mainly based on the law of resistance of a conductor, as shown in Equation (1).

(1) $$R=\rho \frac{l}{A}$$

**Figure 3:**
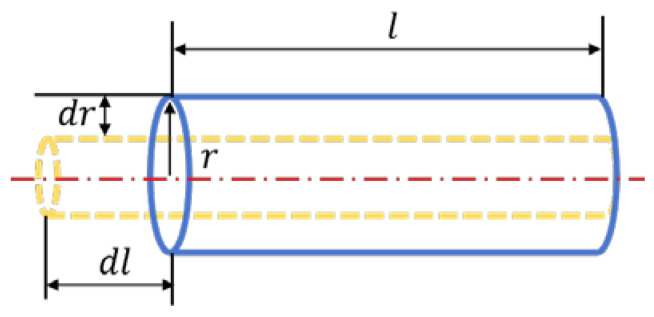
Schematic diagram of conductor deformation under force.

Formula (1) represents the conductor resistance value, where ρ is the resistivity, l is the length, and A is the cross-sectional area. For general conductor selection, which is cylindrical, the cross-sectional area is determined by the radius r. When the conductor undergoes external deformation, as shown in Figure 3, changes in resistivity, length, and radius (i.e., dρ, dl, dr) occur. Consequently, the resistance will also change (i.e., dR).

In use, high-temperature strain gauges are attached to the surface of the measured component. When the component experiences strain, the strain gauges also undergo strain. This strain is transferred, layer by layer, through the transition layer, the substrate, and then to the sensitive grid. According to the law of resistance, the strain generated by the sensitive grid will change the resistance of the sensitive grid, which can achieve the conversion of the strain signal to the resistance signal. Setting dl/l as the strain ε, the relationship between resistance change and strain can be obtained, as shown in Equation (2) [15].

(2) $$\frac{dR}{R}=\left[\left(1+2\mu \right)\varepsilon +\frac{d\rho }{\rho }\right]=K\cdot \varepsilon$$

Equation (2) μ is the Poisson’s ratio of the sensitive grid material, which is the sensitivity coefficient of high-temperature strain gauges, which indicates the resistance variation rate caused by the unit strain.

According to Formula (2), the resistance change is affected by a variety of factors, resulting in the emergence of measurement error, which must be compensated for and corrected, and the specific analysis is as follows.

Firstly, there is the resistance temperature effect. There is a large effect of temperature on the resistivity of metallic materials, as shown in Equation (3). ρ(T) is the resistivity at temperature *T*, $\rho (T_{0})$ is the resistivity at temperature $T_{0}$, and α is the temperature coefficient of resistance (TCR). When α is positive, the resistivity increases with increasing temperature; when α is negative, the resistivity decreases with increasing temperature.

(3) $$\rho (T)=\rho (T_{0})[1+\alpha (T-T_{0})]$$

Secondly, there is a strain transfer error, where the strain is transferred layer by layer from the measured member to the sensitive grids, and there is a strain transfer loss because of the influence of the elastic modulus in the transition layer.

At the same time, there is a thermal output, that is, at a certain temperature, the measured component, the transition layer, the substrate, and the sensitive grids are free from thermal expansion and, due to their different coefficients of linear expansion, the sensitive grids will be affected by the thermal expansion of the measured component to produce strain. This strain is not the strain generated by the force deformation of the mechanical components [16].

**Figure 4:**
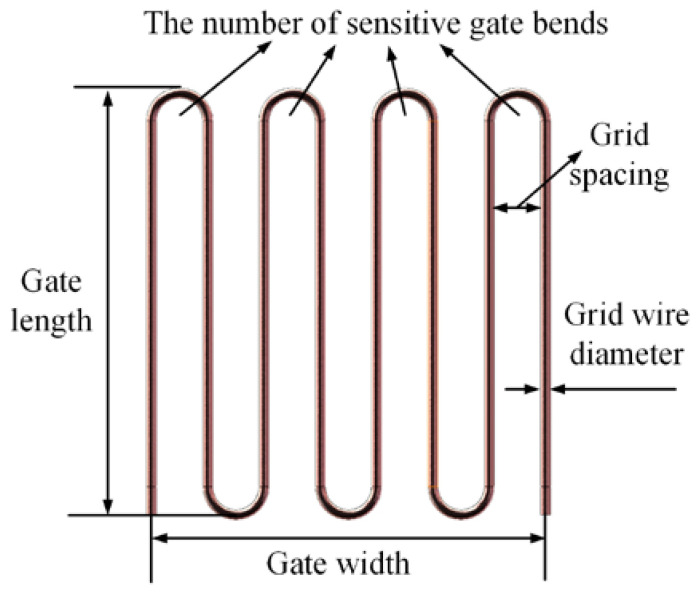
Schematic diagram of sensitive grid structure.

Furthermore, Figure 4 shows a schematic diagram of the structure of the sensitive grids. Among other things, various structural parameters of the sensitive grids also affect the precision of the high-temperature strain gauge measurements, especially the presence of transverse bends.

Therefore, to ensure that the high-temperature strain gauges can be applied at temperatures of more than 800 °C and have high measurement accuracy, it is necessary to do the following.

To ensure that high-temperature strain gauges do not experience phase transformation, high-temperature oxidation damage, or material degradation above 800 °C, the protective layer must provide excellent high-temperature protection, and other structural materials should also withstand high operating temperatures.

Additionally, the sensitive grid should have high and stable resistivity (ρ), a high and stable sensitivity coefficient (K), a stable and near-zero TCR, a near-zero resistive drift rate, and a coefficient of linear expansion equal to or greater than that of the component under test.

Furthermore, high-temperature strain gauges should have low strain transfer error, which is minimized by optimizing the sensitive grid material and structure, as well as the transition layer material and thickness. The transition layer must also exhibit high insulation resistance while ensuring sufficient fatigue strength.

## 4. Research Status

In the following, we sort out the current high-temperature strain gauges applied to temperatures above 800 °C, summarize the breakthroughs, innovations, and shortcomings of the existing research, and provide ideas for the development of high-temperature strain gauges that can be applied in higher temperatures.

### 4.1. Sensitive Grid

As the core element of high-temperature strain gauges, the performance of sensitive grids determines the performance of high-temperature strain gauges. The performance of sensitive grids is determined by the material and structure.

#### 4.1.1. Material

Sensitive grid materials are mainly divided into alloy materials and semiconductor materials. According to the main elements, alloy materials are currently divided into copper-based alloys, iron-based alloys, nickel-based alloys, palladium-based alloys, and platinum-based alloys, as shown in Table 1.

Copper-based alloys and nickel-based alloys are easy to oxidize at high temperatures, resulting in a sharp decline in performance, and are generally not used in the field of high-temperature strain gauges. The sensitive grid materials currently applied to high-temperature strain gauges above 800 °C are mainly iron-based alloys, palladium-based alloys, and platinum-based alloys [18].

The more representative iron-based alloys are various components of ferrochromium aluminum alloys, such as Armour-D and BLC-3 alloys, as shown in Table 1, which can be used for static strain detection at temperatures up to 800 °C. Wang et al. invented a temporary-frame wire-grid high-temperature strain gauge, which was shown to be able to satisfy the static strain measurements at temperatures ranging from room temperature to 1000 °C, in which the sensitive grill material is the ferrochromium aluminum alloy [19]. In the same year, Wang Wenrui et al. calibrated the strain gauge, which had a sensitive grid alloy component of Fe-Cr_25.4_-Al_5.0_, ρ of 1.39 × 10^−6^, TCR of −44 ppm/°C, K of 2.1, and a maximum withstand temperature of up to 1099.85 °C [3]. Xu et al. conducted high-temperature strain measurements on a T4 tie rod in an engine nozzle component using a ZC-NC-G1275-350 high-temperature strain gauge developed by Nuctech, Seattle, WA, USA. The maximum measurement temperature of this model strain gauge is 850 °C [20]. Relevant information shows that the ZC series strain gauge sensitive grid material that is used is iron-chromium aluminum alloy, generally bonded by ceramic adhesive or flame spraying, and the highest measurement temperature can reach 1150 °C. Zhang et al. used the invention of the temporary-frame wire-grid high-temperature strain gauge for strain measurements on turbine blades, and the experimental temperature reached 1042.85 °C [21].

The more representative palladium-based alloys are the various components of palladium-chromium alloys. Palladium-chromium alloys generally limit the elemental chromium content to 13%, resulting in a lower TCR and better high-temperature oxidation resistance. The maximum static operating temperature of this alloy is generally around 800 °C, and the Cr_2_O_3_ generated at high temperatures can provide antioxidant protection and good repeatability of the lift temperature resistance [22]. Liu et al. developed a PdCr thin-film resistive strain gauge on a nickel-based alloy substrate with a measurement temperature of up to 800 °C and a K of around 1.4 [23]. Liu et al. tested the K values of the newly prepared PdCr thin-film strain gauges at various temperatures. The strain coefficients were 2.03 at 600 °C and 2.13 at 800 °C, respectively [24].

Platinum-based alloys generally add tungsten elements to form platinum–tungsten alloys. Platinum–tungsten alloys exhibit a high sensitivity coefficient and can operate at a maximum static temperature of 800 °C or higher. However, the TCR of the alloy is high, which is generally reduced by the heat treatment process. At the same time, Re, Ni, Cr, and other elements are generally added to the platinum–tungsten alloy to improve the comprehensive performance of the alloy. Re, as an alloying element, can improve the tensile strength of the alloy, increase the resistivity, and reduce the TCR; Ni can increase the effect of solid solution strengthening; Cr and rare earth element Y can refine the grain, improve the strength, but, also, Cr and Y can be preferentially oxidized in the substrate surface to form a dense protective layer of oxide film, to inhibit further oxidation within the substrate [1,3]. The modified PtW alloys, such as PtWReNiCrY, can increase the static maximum working temperature to 900 °C [25]. Further improved platinum–tungsten alloys such as PtRhWReZrY can increase the linear interval of the resistance temperature characteristic curve to 1138 °C by the combined effect of fine grain strengthening and diffusion strengthening. In addition, adding molybdenum elements constituting PtRhMoW can further increase the dynamic operating temperature to 1150 °C [17]. Zhang et al. prepared functional thin-film-integrated PtRh6 high-temperature thin-film strain gauges using a combination of photolithography and magnetron sputtering, with a TCR of 88.52 ppm/°C and a K of 1.09 at 900 °C [26]. Liang et al. prepared PtW-PtRh integrated thin-film sensors on Al_2_O_3_ ceramic substrates by magnetron sputtering with annealing. It was shown that the K shifted from 3.82 to 3.68 and from 0 °C to 800 °C, and the sensitivity coefficient was more stable. At 800 °C, the TCR was 823.78 ppm/°C [27]. Chen et al. prepared a high-temperature-resistant PtW thin-film strain gauge using magnetron sputtering on a GH4169 high-temperature alloy substrate with a measurement temperature of up to 600 °C and a strain limit of up to 1400 °C [28].

It can be found, from the above study, that, although alloy materials can achieve high-temperature strain measurement at 800 °C, it is difficult to achieve a breakthrough when it is above 1100 °C because alloys are prone to high-temperature oxidation. Semiconductor materials can withstand higher temperatures, so several high-temperature strain gauges with semiconductor materials as sensitive grids have appeared at home and abroad.

Semiconductor-sensitive gate materials are currently mainly nitride ceramics, silicon-based ceramics, and tin–indium oxide ceramics (ITO).

Nitride ceramics are generally a variety of ceramic compounds prepared by the reaction of metals or metal oxides with nitrogen. Examples include AlN, TaN, TiN, ZrN, TaON, TiAlN, and so on. These materials generally have a high melting point and can be used in the preparation of high-temperature strain gauge-sensitive grids that can be used above 1000 °C. They also have high sensitivity coefficients. The TCR is generally negative, but the nonlinearity problem is more prominent [29]. Chung et al., from the University of Ulsan, Korea, prepared a TaN thin-film strain gauge by annealing at 900 °C for 1 h with a TCR of −84 ppm/°C and a K value of 4.12 [30].

Silicon-based ceramics are generally silicides, silicates, or other silicon-containing compounds synthesized from the element silicon with other elements. Examples include BTi_2_, TiSi_2_, TaSi_2,_ and WSi_2_. These materials have a high melting point and good high-temperature oxidation resistance. However, the low sensitivity coefficient and high TCR of these materials limit the application in high-temperature strain applications. Wu et al. developed TiB_2_/SiCN thin-film strain gauges on substrates made of nickel-based alloys, which could reach a maximum operating temperature of 700 °C with a K of 7.12 in the absence of deposition of antioxidant protective layer [31]. Xu et al. employed Direct Ink Writing and Laser Scanning (DIW-LS) to create an ITO/PDC film capable of withstanding temperatures of up to 1250 °C and with a K value of −6.0 [32].

Tin–indium oxide ceramics (ITO) are generally compounds composed of indium oxide (In_2_O_3_) and tin oxide (SnO_2_) in a certain ratio. For example, In_2_O_3_:SnO_2_ (90:10 wt%). These materials have better high-temperature stability and sensitivity coefficients. Yang constructed an in situ self-compensating Pt-ITO thin-film strain gauge featuring a nanolaminate structure, which can be tested at temperatures of up to 1200 °C, with a TCR of 30 ppm/°C and a K value of 10 [33]. Gregory et al. developed a Pt-ITO with a compositional ratio of 11.2 wt% ITO and 88.8 wt% Pt, which was tested at temperatures from room temperature to 1200 °C with a TCR of -79 ppm/°C and a K of 26 [34].

**Figure 5:**
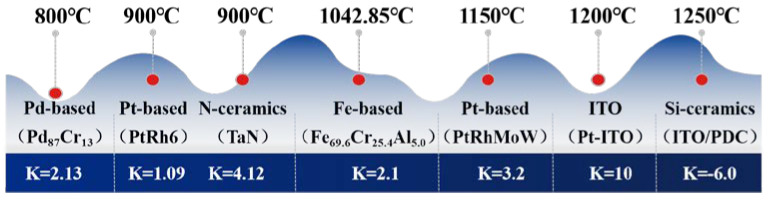
Operating temperature limits for different sensitive grid materials [17,20,21,24,26,30,32,33].

In summary, the sensitive grid materials suitable for high-temperature strain gauges above 800 °C are presented in Figure 5. Pd_87_Cr_13_ offers good high-temperature stability, strong corrosion resistance, a stable resistance-temperature coefficient, and a lower cost compared to PtRh6 and PtRhMoW. However, its upper-temperature limit is relatively low. When operating above 900 °C, its coefficient of thermal expansion is high, which significantly reduces both its high-temperature stability and measurement accuracy. PtRh6 and PtRhMoW exhibit excellent high-temperature stability, with the addition of Rh significantly improving the antioxidant performance of the sensitive grids. However, due to the use of precious metals, such as Pt and Rh, the cost is relatively high. Similarly, TaN possesses a very high melting point and outstanding high-temperature resistance and corrosion resistance, but its cost is also relatively high. Fe_69.6_Cr_25.4_Al_5_ demonstrates good high-temperature performance. The presence of Cr and Al provides antioxidant protection, and its low coefficient of thermal expansion enhances the accuracy of the strain gauge. However, compared to PtRh6, Fe_69.6_Cr_25.4_Al_5_ has relatively lower precision, stability, and corrosion resistance. Nevertheless, due to its lower cost, Fe_69.6_Cr_25.4_Al_5_ offers a high cost–performance ratio and commercial value. Pt-ITO and ITO/PDC materials provide superior high-temperature stability, enabling operation at higher temperatures. However, compared to Fe_69.6_Cr_25.4_Al_5_, both Pt-ITO and ITO/PDC are more costly, and their mechanical strength is lower, leading to reduced fatigue life in environments subject to mechanical stress.

At present, few papers have been published by non-Chinese groups on high-temperature strain gauges applicable to temperatures above 800 °C. The related research mainly focuses on the influence of new materials and nanotechnology on the comprehensive performance of high-temperature strain gauges, and the testing temperature mainly focuses on about 500 °C. Zarfl et al. investigated the preparation process and performance parameters of TiAlN_X_O_1−X_ high-temperature thin-film strain gauges and, finally, prepared thin-film strain gauges with the best performance in an oxygen-free mixture atmosphere. The strain gauges possessed high-temperature stability at 500 °C with K of 2.4 [35]. Rahman et al. fabricated a silver (Ag) nanofilm-type strain gauge by an aerosol injection method along with heat treatment. The K was 3.15 under the 500 °C test, which is higher than that of commercial strain gauges at the same operating temperature. The reason for this was analyzed to be due to the fact that the sintered nanoparticles increase the porosity of the film, which enhances the Poisson’s ratio of the film and, hence, the K value [36]. A thin-film strain gauge consisting of 7 nm AlN and 3 nm Pt was designed by Schmid et al. by utilizing an annealing treatment at 900 °C under an Ar gas environment for 1 h. The mixing of AlN and Pt layers was achieved to enhance the electrochemical stability of the strain gauge. Compared with pure Pt film strain gauges of the same thickness, it has the same K, but the TCR is reduced by 3–4 times. It also possesses good stability in a 500 °C air environment [37].

In recent years, high-temperature strain gauge technology has evolved significantly within the commercial sector, with several major companies offering a diverse range of products. StrainSense, based in the Old Stratford, UK, has introduced the HFN series (NiCr alloys), which is capable of operating at temperatures of up to 870 °C, while the HFP series (PtW alloys) is designed for applications reaching 1038 °C. Micro-Measurements, a leading company in the Wendell, NC, USA, offers the ZWP series (PtW alloys), with a maximum operating temperature of 1038 °C, and the ZC series (Fe-Cr-Al alloys), which can withstand temperatures of up to 1150 °C. HPI also provides NiCr alloys that can operate at a maximum temperature of 1038 °C. Additionally, HPM (Pleasant Hill, CA, USA), another prominent U.S. manufacturer, produces the STN series (NiCr alloy) with a maximum temperature rating of 900 °C, while both the STF series (Fe-Cr-Al alloy) and STP series (PtW alloy) can withstand temperatures of up to 1150 °C. These findings further validate that the materials examined above can be successfully applied in practical testing scenarios. Jan et al., on the use of a thermal spraying process on the surface of the workpiece-mounted Micro-Measurements, Inc. (Wendell, NC, USA), produced ZC-NC-G1262-120 strain gauges the heat resistance of which was found to be as high as 1000 °C and, in 500 °C strain measurements, the results were good [38]. NASA GRC prepared a high-temperature thin-film strain gauge using PdCr as the sensitive grid material. The strain gauge was applied to an aero-engine combustor device. Experimental tests were conducted at 1100 °C with better results. This proves that the PdCr material can be applied to higher temperatures [39].

#### 4.1.2. Structure

In addition to the sensitive gate material, the structure of the sensitive gate also has a significant impact on the performance of high-temperature strain gauges. In recent years, researchers have studied the relationship between structure and measurement accuracy.

Among them, the wire-sensitive grid is mainly made of metal wire wound into a grid structure, as shown in Figure 4. It mainly contains the following five structural parameters:

① Size: gate length ($l_{A}$) × gate width ($l_{B}$);
② Grid wire diameter (d);
③ Grid spacing (h);
④ the number of sensitive gate bends ($N_{S}$).

**Figure 6:**
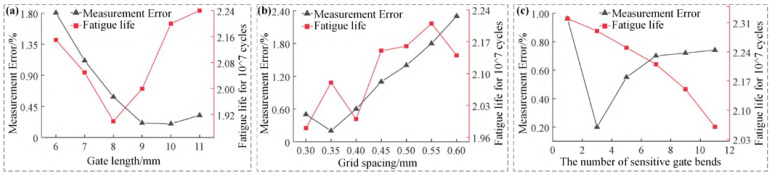
Influence of structural parameters on measurement error and fatigue life [41]. (**a**) Measurement error and fatigue life at different grid lengths, (**b**) Measurement error and fatigue life at different grid spacing, (**c**) Measurement error and fatigue life at different the number of sensitive gate bends.

The above structural parameters have different impacts on the performance of high-temperature strain gauges. Wang et al. utilized the finite-element method to simulate and test the self-designed free-frame wire-grid high-temperature strain gauges and came up with the following conclusions: the smaller the d (0.02–0.04 mm), the higher the measurement accuracy; the optimal value exists between the h (0.1–0.6 mm), the $l_{A}$ (2–10 mm), and the measurement accuracy. Other authors optimized the structural parameters and reduced the measurement error of high-temperature strain gauges to 13.2% [16]. Yu et al. also analyzed the effect of the structural dimensions of the sensitive grids on measurement accuracy by using finite element software (Ansys19.0). It was found that the smaller d (0.01–0.05 mm), the higher the measurement accuracy; the larger h (0.1–0.6 mm), the greater the measurement precision; the longer $l_{A}$ (2.5–6.5 mm), the greater the measurement accuracy [40]. Ai et al. developed a simply supported beam-measurement error model and a cantilever beam-fatigue life model, respectively, in order to investigate the influencing factors of the two objectives, measurement error and fatigue life. As shown in Figure 6, the lower d (0.02–0.04 mm), the greater the measurement accuracy and the lower the fatigue life; h (0.3–0.6 mm), $l_{A}$ (6–11 mm), and the optimal value between the measurement accuracy and the fatigue life; $N_{S}$ (1–11) and the measurement accuracy of the optimal value; the fatigue life with the increase in $N_{S}$ is a decreasing trend [41].

In the above study, although the material parameters of the selected sensitive grids are different and the other fixed value parameters are different when studying a certain parameter, the overall law is roughly similar. However, the strength of the influence of each structural parameter on the measurement accuracy and the interaction between them were not further analyzed.

Zhang et al. further investigated the above issue. As shown in Table 2, by increasing a certain structural parameter variable, changes in the measurement accuracy and fatigue life, the two objective parameters, were observed. On this basis, the value of another structural parameter variable was adjusted to analyze its impact on the relationship between the first structural parameter variable and the objective parameters. In Table 2, another structural parameter is generally taken as follows. h is generally taken as 0.35 mm, 0.4 mm, and 0.45 mm; $N_{S}$ is generally taken as 3, 5, and 7; $l_{A}$ is generally taken as 7 mm, 8 mm, and 9 mm [42].

The above study confirmed the existence of interactions between structural parameters. Zhang et al. further established response surface models for measurement accuracy and fatigue life and investigated the strength of the impact of different structural parameter interactions on measurement accuracy and fatigue life. The study found that the combined effect of h and $l_{A}$ had the strongest impact on measurement accuracy, while the combined effect of h and $N_{S}$ had the weakest impact on measurement accuracy. In contrast, the combined effect of h and $N_{S}$ had the strongest impact on fatigue life, while the combined effect of $l_{A}$ and $N_{S}$ had the weakest impact on fatigue life [42].

**Figure 7:**
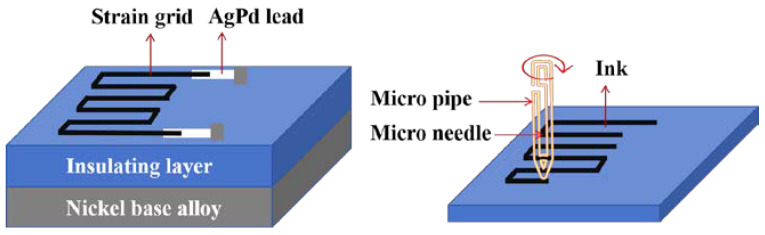
Thin-film strain gauge structure and schematic of sensitive grid printing [31].

Thin-film-type sensitive grids are mainly generated directly on the substrate using vacuum deposition or sputtering methods, as shown in Figure 7. Therefore, in addition to the basic configuration similar to the filament-type sensitive grids, more complex structures can be processed [31].

**Figure 8:**
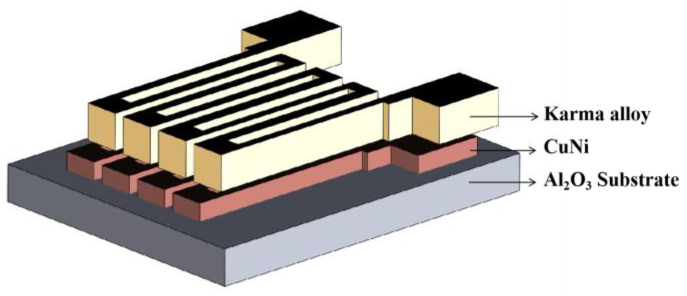
Serpentine double-layer thin-film strain gauges [43].

Zhang et al. fabricated a Karma-CuNi serpentine bilayer thin-film strain gauge with an integrated temperature self-compensation function. Meanwhile, it was found that the TCR could be adjusted by adjusting the sensitive gate of the Karma layer and the sensitive gate of the CuNi layer. Meanwhile, it was realized that the TCR of the strain gauge could be modulated to 0 ppm/°C by annealing the strain gauge at 200 °C with a K of about 2.3 under the thickness ratio of about 2.5:1, which provided a solution to the problem of the larger TCR at high temperatures, as shown in Figure 8 [43].

**Figure 9:**
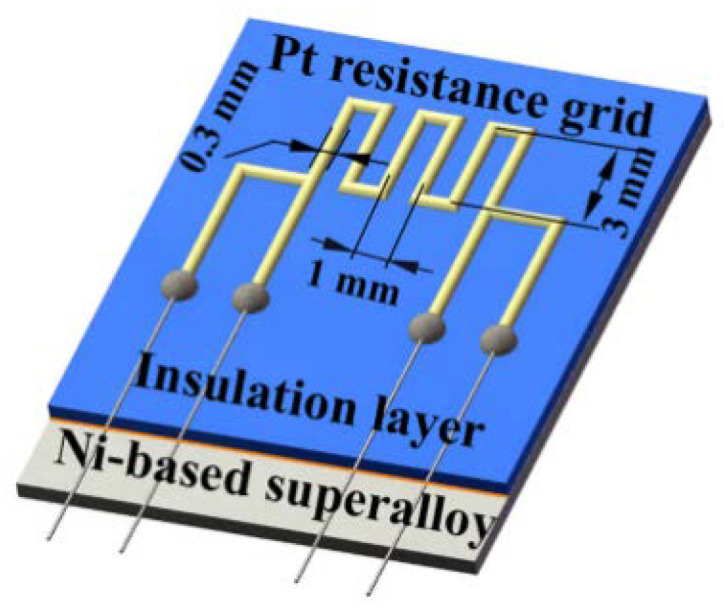
Thin-film strain gauges with four-wire structured Pt [44].

Wu et al. prepared a thin-film sensitive gate with a four-wire structure, as shown in Figure 9. The thickness was 15 um, the line width was 0.3 mm, and the gate spacing was 1 mm. This structure can improve measurement accuracy by reducing the errors introduced by contact resistance and wire resistance. The high-temperature strain gauges were tested to have K of 1.26 and 1.09 at 700 °C and 800 °C, respectively; at 800 °C, the TCR was 1730 ppm/°C [44].

**Figure 10:**
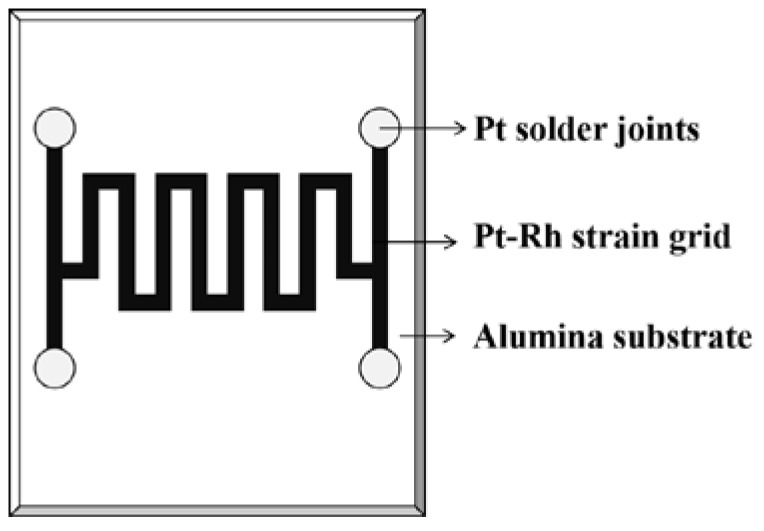
Thin-film strain gauges with four-wire structured Pt-Rh [45].

Zhao et al. designed a thin-film strain gauge with a four-wire structure, as shown in Figure 10. Among them, the sensitive grids were printed with a mixed ink of Pt paste and Rh powder with glass phase and metal–metal alloy synergies. It was tested to work properly at 1100 °C when K was about 1.5 [45].

**Figure 11:**
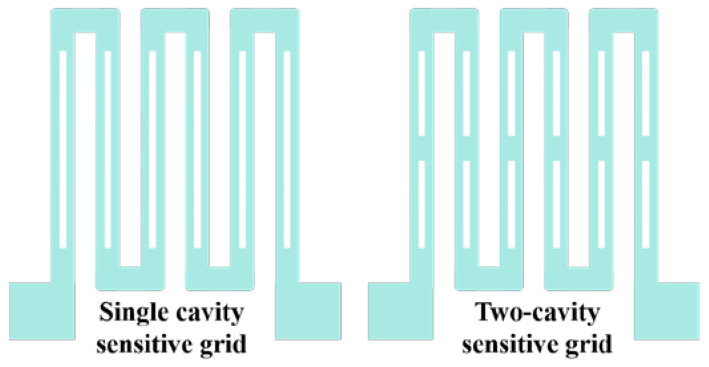
Sensitive grids with cavity structure [46].

Zhao et al. optimized the design of two thin-film sensitive grids with a cavity structure. Tests show that the design of the cavity structure can effectively increase the strain gauge sensitivity coefficient K. As shown in Figure 11, the left single-cavity structure has a strain gauge K value of 1.92, and the right double-cavity structure has a strain gauge K value of 1.91 [46].

**Figure 12:**
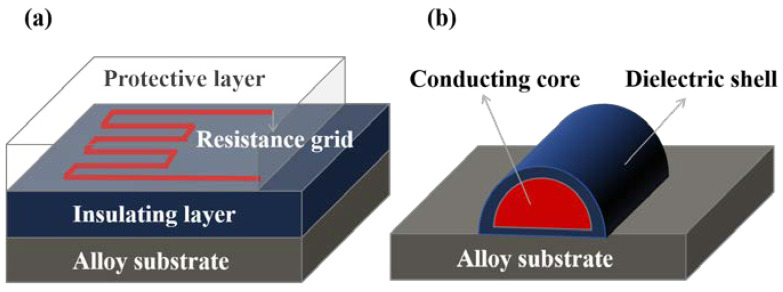
Two types of thin-film high-temperature strain gauges: (**a**) laminated structure strain gauges; (**b**) core–shell structure strain gauges [47].

Wu et al. manufactured a high-temperature thin-film strain gauge with a core–shell structure in situ on an alloy component using a coaxial multi-ink printing technique, as shown in Figure 12b. Direct deposition of the coaxial material onto the structure’s surface was performed, with the structure mainly featuring a Pt core for strain measurement and a dielectric shell for electrical isolation and temperature protection. Compared to the conventional laminated thin-film type strain gauges shown in Figure 12a, the strain gauges fabricated by this process have excellent high-temperature performance, with a resistance change of only 0.08%/h at 800 °C, and can withstand thermal shock [47].

### 4.2. Transition Layer

The transition layer is used to bond the sensitive grid to the substrate or the surface of the structure under test. This requires that the transition layer has both a high bond strength and a high insulation resistance to avoid interference with the sensitive grid signal. Therefore, the transition layer is often further subdivided into insulating and bonding layers.

**Figure 13:**
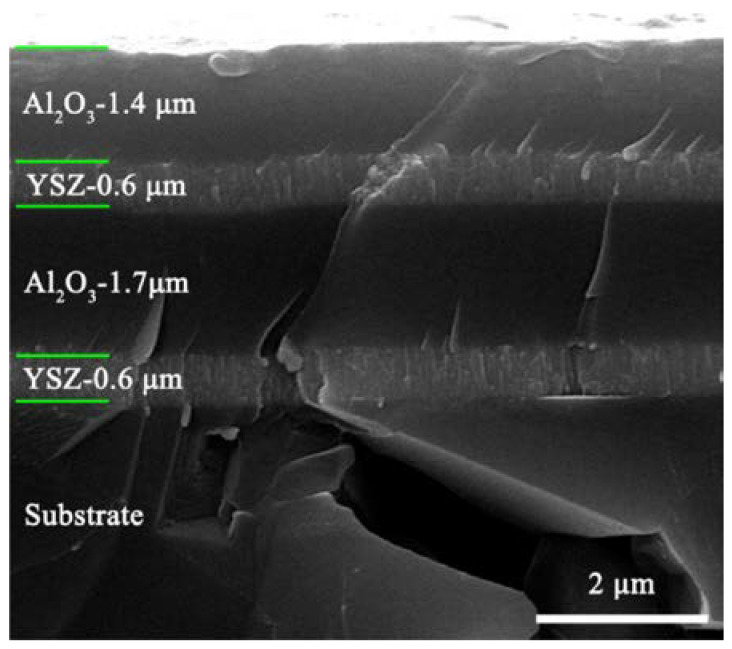
A thin-film high-temperature strain gauge cross-section SEM morphology [49].

Yang et al. prepared a transition layer consisting of a YSZ/Al_2_O_3_ insulating layer and a NiCrAlY bonding layer on the surface of a nickel-based high-temperature alloy, and the insulation resistance was higher than 150 kΩ at 800 °C, which could meet the testing needs [48]. Liu et al. created a YSZ/Al_2_O_3_/YSZ/Al_2_O_3_ four-layer insulating layer, as shown in Figure 13, with an insulation resistance of about 200 kΩ at 800 °C [24,49].

Chen et al. prepared two composite insulating layers, Al_2_O_3_ sol/Al_2_O_3_ mixture and Al_2_O_3_/Si_3_N_4_, which were tested at 1200 °C with insulation resistances of 51 kΩ and 54 kΩ, respectively, whereas the resistance of the insulating layer prepared only from Al_2_O_3_ mixture was only 38 kΩ, which proved that the composite insulating layer could effectively enhance the insulating performance [50]. Peng et al. created an Al_2_O_3_/CeO_2_/Al_2_O_3_ composite structural insulating layer with excellent insulating properties and an insulation resistance as high as 1.9 MΩ at 900 °C [51].

### 4.3. Protective Layer

The protective layer is mainly to isolate the sensitive grids from the external harsh environment to avoid high-temperature oxidation and erosion failure of the sensitive grids, which plays an important role in increasing the working temperature of the strain gauges. As an important part of high-temperature strain gauges, researchers have also been exploring protective layers for higher temperatures in recent years.

**Figure 14:**
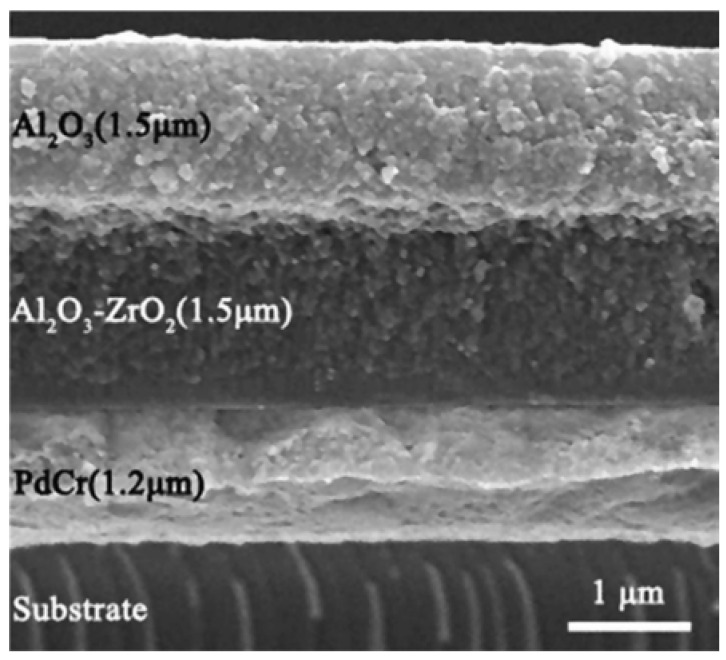
A thin-film high-temperature strain gauge cross-section SEM morphology [52].

Yang et al. designed a 3 μm thick Al_2_O_3_/ZrO_2_ protective layer on the surface of PrCr-sensitive grids by DC reactive sputtering, and the strain gauges can work properly at temperatures of up to 700 °C [48]. Liu et al. constructed a heterogeneous Al_2_O_3_-ZrO_2_/Al_2_O_3_ ceramic protective layer. As shown in Figure 14, compared with single-layer Al_2_O_3_ and Al_2_O_3_-ZrO_2_, it has the advantages of dense structure, fewer defects, and no cracks at the interface at a high temperature of 800 °C, and the high-temperature protection ability is better, as shown in the SEM morphology analysis and XRD mapping analysis [52].

**Figure 15:**
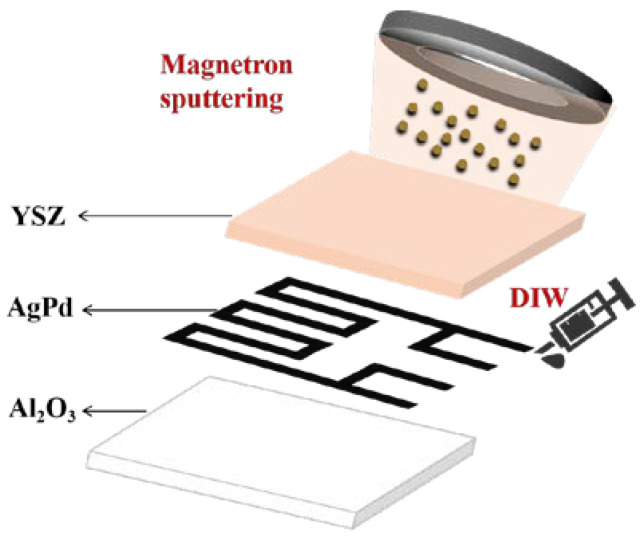
AgPd thin-film strain gauges with YSZ as a protective layer [53].

Zhao et al. investigated the effect of YSZ as a protective layer on AgPd-sensitive grids at high temperatures, as shown in Figure 15. It was found that the resistance drift of the strain gauges sprayed with 2 μm thicknesses of YSZ protective layer was reduced from 0.29%/h to 0.04%/h at 800 °C for 8 h compared to the strain gauges without the protective layer [53].

**Figure 16:**
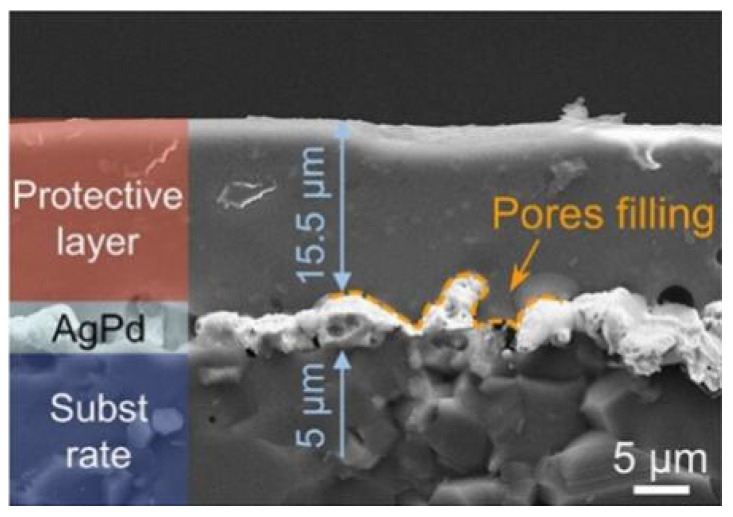
Strain gauges with a microcrystalline glass–ceramic protective layer [54].

Zeng et al. created a microcrystalline glass–ceramic protective layer and applied it to the high-temperature protection of AgPd-sensitive grids. Experiments showed that the microcrystalline glass protective layer had a high density and no film defects after sintering, as shown in Figure 16. After sintering, the protective layer fills the pores within the AgPd sensitive layer and combines with the glass phase in the sensitive layer to form a continuous solid solution, which, in turn, prevents the particles from volatilization, agglomeration, and oxidation and has excellent high-temperature protection properties [54].

**Figure 17:**
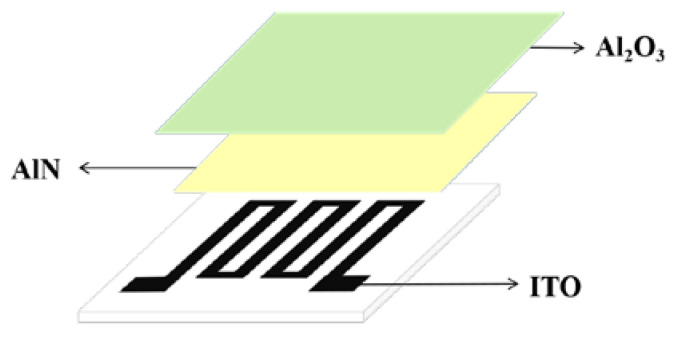
Thin-film strain gauges with AIN/AI_2_O_3_ protective layer [55].

Li et al. explored the effect of the AIN/AI_2_O_3_ thin-film protective layer on the high-temperature performance of ITO thin-film strain gauges. It was found that the protective layer was tightly adhered to the layers between the sensitive grids without any gap, which effectively suppressed the oxygen diffusion and enhanced the high-temperature stability of the ITO thin-film strain gauges, as shown in Figure 17. The resistance drift rate was only 1.55%/h for 8 h at 1100 °C [55].

### 4.4. Preparation Process

The fabrication process directly determines the final performance of high-temperature strain gauges. This section reviews and analyzes the fabrication processes for high-temperature strain gauges used at temperatures above 800 °C, with a focus on the process flows for wire-type and thin-film strain gauges.

The fabrication process of wire-type strain gauges typically involves four steps: alloy wire preparation, sensitive grid winding, encapsulation and protection, and post-processing. Alloy wire preparation generally requires three processes: vacuum arc melting, forging, and drawing. This step determines the material composition of the sensitive grid and the diameter of the grid wire, which are critical to the performance of the strain gauge. The sensitive grid winding involves winding the alloy wire into the designed grid structure. The encapsulation and protection process uses plasma spraying to form the insulation and protective layers of the strain gauge, while the grid is connected to the lead wires by brazing. The post-processing step involves high-temperature annealing in an inert atmosphere to enhance the stability of the strain gauge [1]. In summary, the advantage of wire-type strain gauges lies in their ability to ensure stable resistance values for the sensitive grid, making them more suitable for mass production and industrial applications. However, their size is difficult to miniaturize, with a gate length typically greater than 5 mm, which makes it challenging to achieve precise measurements of local strain.

**Figure 18:**
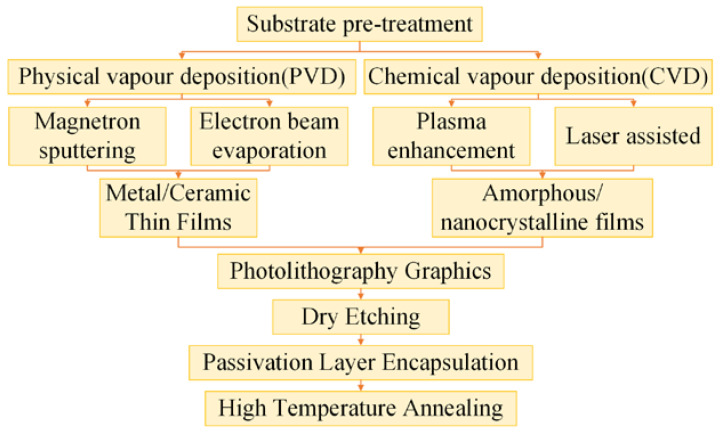
Conventional thin-film deposition processes.

The fabrication process of thin-film strain gauges offers multiple technical paths. The traditional thin-film deposition process, as shown in Figure 18, consists of physical vapor deposition (PVD) and chemical vapor deposition (CVD). Thin films produced by PVD are highly dense, and the patterning precision can reach the micron level. However, PVD has high equipment costs and long processing cycles [53]. CVD technology allows for uniform coverage of complex surfaces and flexible control of material composition, but it requires high-temperature processes and poses safety risks with the precursor materials. The patterning precision of CVD is typically lower than that of PVD [56].

**Figure 19:**
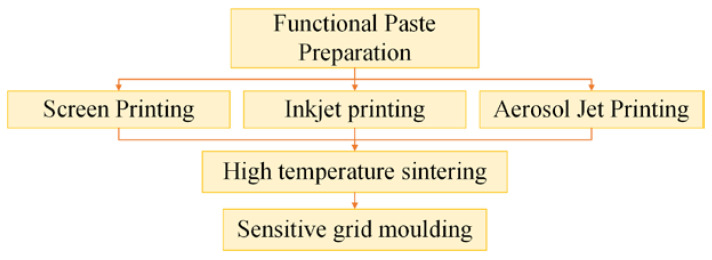
New thin-film printing process.

The new thin-film printing processes, as shown in Figure 19, include screen printing, inkjet printing, and aerosol jet printing. Screen printing offers a large printing area per cycle, enabling low-cost mass production while allowing precise control over the printing thickness. However, its resolution is limited, making it difficult to achieve micron-level fine pattern printing [57]. Inkjet printing technology provides a high degree of freedom, enabling the direct digital printing of complex patterns. However, it suffers from poor high-temperature performance, and the printed films are prone to oxidation and failure in high-temperature environments [58]. Aerosol jet printing offers ultra-high precision, is capable of fine and curved surface printing, and has a wide range of compatible inks, including metal/ceramic nanoparticles. However, it is characterized by high equipment costs and slow production speeds, making it unsuitable for large-scale manufacturing [59].

### 4.5. Future Outlook

The latest research shows that the high-temperature strain gauge technology has basically been able to be used for strain detection at about 800 °C, but further improvement is still needed for both detection accuracy and fatigue life. In addition, the latest data show that the operating temperature of the new generation of aero-engine is up to 2000 °C [60], and the temperature of the turbine disc is also around 1500 °C [17]. The existing high-temperature strain gauge technology is not enough to support the new aero-engine pre-research test and strain detection needs, and there is an urgent need to improve the operating temperature.

Combined with the existing research, in order to further improve the operating temperature of high-temperature strain gauges in the future, it is necessary to start from the material and structure aspects and improve the high-temperature resistance of high-temperature strain gauges in all aspects.

**Figure 20:**
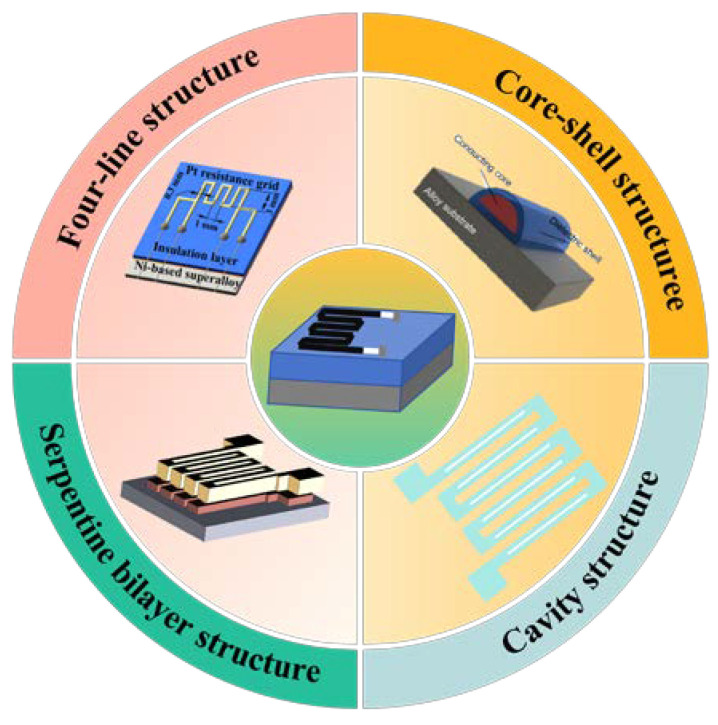
Special structures for sensitive grids [31,43,44,46,47].

(1) It is necessary to continue to study how to improve and optimize the performance of the sensitive grids of high-temperature strain gauges. Currently, the highest alloy material sensitive gate can be used for 1150 °C, but, at higher temperatures, changes will occur in the alloy material’s internal lattice, as well as other changes, resulting in a sharp decline in alloy performance and instability, making it difficult to achieve further breakthroughs. Semiconductor materials, such as nitride ceramics, silicon-based ceramics, and tin–indium oxide ceramics, have been proven to be sensitive as a strain gauge gate and work properly at higher temperatures; one of the silicon-based ceramics, ITO/PDC, when used as a sensitive gate prepared by high-temperature strain gauges, has been successfully used in 1250 °C under strain detection. In the future, semiconductor materials will become a breakthrough for strain-sensitive grids to improve the operating temperature. At the same time, it is essential to overcome the limitations of existing single-material systems. Referencing the thin-film strain gauge design based on silver nanoparticles by Rahman et al. [36], future research could explore embedding sub-micron metal pathways (such as TaC nanoparticles) within semiconductor materials. Under high-temperature conditions, the metallic phase would form a percolation network, enabling automatic compensation for thermally-induced resistance drift and reducing the temperature coefficient of resistance (TCR) [61,62].

(2) It is found that the optimized design of the basic structural parameters of the sensitive grids can improve the measurement accuracy and fatigue life of the high-temperature strain gauges. For example, the response surface method can be used to study the effect of different combinations of structural parameters on the performance of sensitive grids, and the multi-objective optimization algorithm can improve the overall performance of sensitive grids. In addition, the special structure of sensitive grids can also help to improve the detection performance of strain gauges. Existing research in the innovative development of four-wire structures, core–shell structures, serpentine double-layer structures, and cavity structures can effectively improve the measurement performance of high-temperature strain gauges, such as the operating temperature, sensitivity coefficient, resistance temperature coefficient, and so on, as shown in Figure 18. Future designs of sensitive gate structures could explore the development of a multi-scale collaborative optimization system. At the macroscopic level, bio-inspired fractal structures based on topology optimization can improve strain transfer efficiency and reduce thermal stress concentration [63]. At the mesoscopic level, referring to the core–shell structure shown in Figure 20, atomic layer deposition (ALD) technology can be used to construct a nanometer-scale interfacial transition layer, achieving synergistic enhancement of the semiconductor’s high sensitivity and the metal’s stable conductivity [47,64]. At the microscopic level, an asymmetric radial lattice layout, aided by finite element design combined with strain–temperature field decoupling algorithms, can reduce thermal output errors.

(3) It is important to continue to study the transition layer materials and structures suitable for use at higher temperatures. One method is to improve the insulation resistance of the transition layer in the high-temperature environment, and the second is to improve the bond strength of the transition layer in the high-temperature environment. Existing research has found that multi-layer composite structures of different materials can effectively improve the insulation resistance; using the same material sol and mixture of composite preparation of the transition layer also helps to improve the insulation resistance.

(4) It is important to continue to study the protective layer materials and structures suitable for use at higher temperatures. The main goal of this research is to optimize the protective layer to reduce the same temperature and loading time under the strain gauge resistance drift rate. The protective layer is generally made of semiconductor materials, which can be strengthened by multilayer composite and solid solution strengthening with sensitive grids under high-temperature environments to improve the protection performance. In the future, intelligent, protective layers with self-sensing capabilities could be designed. For instance, a distributed optical fiber grating array could be embedded in an Al_2_O_3_-YAG eutectic protective layer to achieve in situ monitoring of both temperature and strain [65]. Magnetron sputtering could be used to fabricate TiAlN/MoSi_2_ multilayer films, introducing controllable residual compressive stress through interface stress engineering, which significantly inhibits crack initiation during high-temperature oxidation [66,67]. Additionally, integrated miniature thermoelectric generators could convert waste heat into electrical energy to power active cooling microchannels, thereby forming an active defense mechanism [68].

(5) In addition to the high-temperature strain gauge technique, there are other types of high-temperature strain detection techniques, as shown in Table 3. The principle of the optical fiber method is based on fiber grating or distributed sensing, where the strain is measured by the change in optical signal. Li et al. designed a fiber vernier harmonic sensor based on a parallel dual-fiber Fabry-Perot interferometer (FPI), which can perform strain measurements at 900 °C [69]. Mathewze, by pre-compressing the Fiber Bragg Grating (FBG) achieved a strain measurement at 800 °C [70]. However, at high temperatures, the temperature sensitivity of optical fibers is much higher than the strain sensitivity, which necessitates complex temperature compensation; so, optical fiber strain detection technology at higher temperatures needs further research. The laser speckle method is used to make strain measurements by analyzing the changes in the speckle pattern formed by laser irradiation of an object’s surface. Kaczmarek et al. carried out strain and crack measurements on a ceramic surface at 1200 °C by laser speckle method [71]. Digital image correlation (DIC) is used to measure strain by analyzing the displacement field on the surface of an object through an image-matching algorithm. Rossmann et al. proposed an in situ digital image (DIC) with synchrotron measurements, which successfully achieved strain measurements at 870 °C [72]. These two methods enable full-field non-contact strain measurements but require a stable light source and are susceptible to ambient light. Surface Acoustic Wave (SAW) sensors are used to measure strain by the change of propagation velocity on the acoustic surface. Zhang et al. successfully achieved strain measurement at 500 °C using surface acoustic wave (SAW) sensors [73]. However, the complex manufacturing process and high cost of Surface Acoustic Wave (SAW) sensors limit their range of applications.

High-temperature strain gauge measurement techniques can be combined with other high-temperature strain detection technologies, such as non-contact strain measurement technology, to achieve online joint measurement and improve measurement accuracy and operating temperature so as to solve the high-temperature strain detection problems at higher temperatures. High-temperature strain gauge technology can be integrated with Digital Image Correlation (DIC) technology to achieve complementary verification of multi-scale data. High-temperature strain gauges can be installed at critical stress concentration regions to provide local high-precision dynamic strain data, while DIC technology simultaneously captures the full-field strain distribution. The time-domain accuracy of the strain gauges is used to correct the low-frequency errors in DIC, and the full-field data from the DIC are employed to correct the installation position deviations of the strain gauges. By fusing the data obtained from both techniques, strain measurement errors can be effectively reduced [71,72]. Additionally, high-temperature strain gauge technology can be coupled with infrared thermography and Acoustic Emission (AE) technology for integrated monitoring. High-temperature strain gauges monitor the accumulation of local plastic strain, while infrared thermography inversely reconstructs the stress field through the thermoelastic effect and cross-validates with strain gauge data. Simultaneously, AE technology is utilized to capture transient events such as strain gauge delamination or material cracking. The coupling of these three physical fields enables intelligent monitoring of the tested components [74,75].

## 5. Conclusions

This paper systematically investigates high-temperature strain gauges applied at temperatures above 800 °C. It begins by summarizing the measurement principles of high-temperature strain gauges, analyzing the sources of measurement errors, and discussing strategies to improve measurement accuracy. Next, this paper provides an overview and analysis of the materials and structures currently used in high-temperature strain gauges operating at temperatures of 800 °C and above, focusing on the sensitive grids, transition layers, and protective layers. Finally, based on current research, this paper offers ideas and directions for the future development of high-temperature strain gauges and detection technologies for use at even higher temperatures.

## Figures and Tables

**Table 1:** Commonly used sensitive grid alloys [1,17].

Alloys	Components	Sensitivity Factor (K)	TCR/(ppm/°C)	Static Maximum Temperature/°C	Reference
Cu-based	Cu_60_Ni_40_	2.0	20	200	[1]
Fe-based	Fe_70_Cr_20_Al_10_	1.99	−110	800	[1]
	Fe_67.5_Cr_25_Al_7.5_	2.5	28	800	[1]
Ni-based	Ni_80_Cr_20_	2.0	110	400	[1]
	Ni_73_Cr_20_Al_7_	2.0	10	400	[1]
Pd-based	Pd_87_Cr_13_	1.7	142	800	[1,17]
Pt-based	Pt_92_W_8_	4	248	800	[1,17]
	Pt_100_	3.88	400	1000	[1]

**Table 2:** Influence of structural parameters on measurement accuracy and fatigue life [42].

Changes in Structural Parameters	Variation of Target Parameters (Measurement Accuracy/Fatigue Life)	Another Structural Parameter Affects
$l_{A}$ (5–11 mm) increase	Measurement accuracy decreases and then increases	h and $N_{S}$ affect the $l_{A}$ value of the maximum value of measurement accuracy.
h (0.3–0.6 mm) increase	Measurement accuracy decreases	$\mathrm{Different}\;l_{A}$ affects the rate at which measurement accuracy decreases with increasing h.
$N_{S}$ (1–11) increase	Measurement accuracy decreases and then levels off	$\mathrm{Different}\;l_{A}$$\;\mathrm{affects}\;\mathrm{the}\;\mathrm{rate}\;\mathrm{at}\;\mathrm{which}\;\mathrm{measurement}\;\mathrm{accuracy}\;\mathrm{decreases}\;\mathrm{with}\;\mathrm{increasing}\;N_{S}$.
$l_{A}$ (5–11 mm) increase	$\mathrm{The}\;\mathrm{fatigue}\;\mathrm{life}\;\mathrm{first}\;\mathrm{decreases}\;\mathrm{and}\;\mathrm{then}\;\mathrm{increases},\;\mathrm{and}\;\mathrm{the}\;\mathrm{fatigue}\;\mathrm{life}\;\mathrm{change}\;\mathrm{is}\;\mathrm{no}\;\mathrm{longer}\;\mathrm{obvious}\;\mathrm{after}\;l_{A}$ reaches 10 mm.	
h (0.3–0.6 mm) increase	$\mathrm{Fatigue}\;\mathrm{life}\;\mathrm{is}\;\mathrm{increasing}\;\mathrm{when}\;l_{A}$$\;\mathrm{is}\;8\;\mathrm{mm};\;\mathrm{fatigue}\;\mathrm{life}\;\mathrm{is}\;\mathrm{increasing}\;\mathrm{and}\;\mathrm{then}\;\mathrm{decreasing}\;\mathrm{when}\;l_{A}$$\;\mathrm{is}\;7\;\mathrm{and}\;9\;\mathrm{mm};\;\mathrm{fatigue}\;\mathrm{life}\;\mathrm{is}\;\mathrm{increasing}\;\mathrm{when}\;N_{S}$$\;\mathrm{is}\;3\;\mathrm{and}\;5;\;\mathrm{fatigue}\;\mathrm{life}\;\mathrm{is}\;\mathrm{increasing}\;\mathrm{and}\;\mathrm{then}\;\mathrm{decreasing}\;\mathrm{when}\;N_{S}$ is 7.	
$N_{S}$ (1–11) increase	$\mathrm{The}\;\mathrm{fatigue}\;\mathrm{life}\;\mathrm{decreases}\;\mathrm{continuously}\;\mathrm{for}\;l_{A}$$\;\mathrm{of}\;7\;\mathrm{and}\;8\;\mathrm{mm};\;\mathrm{for}\;l_{A}$ of 9 mm, the fatigue life increases and then decreases.	

**Table 3:** Comparison of other high-temperature strain detection techniques.

Technology	Advantages	Disadvantages	Application	Reference
Optical Fiber Method	EMI-resistant, multi-point measurement, corrosion-resistant.	Significant temperature effects, complex temperature compensation required, poor stability at high temperatures.	Electromagnetic interference environment, low strain detection	[69,70]
Laser Speckle Method	Full-field measurement, non-contact	Requires stable light source, complex data processing, low repeatability	Dynamic strain on flat surfaces	[71]
Digital Image Correlation (DIC)	Non-contact, full-field measurement, 3D reconstruction	Requires surface texture, light-sensitive, limited to surface strain	Surface strain under visible light (<800 °C)	[72]
Surface Acoustic Wave (SAW)	Wireless/passive, high-temp resistant (up to 1200 °C)	Complex fabrication, difficult calibration, high cost	Rotating components	[73]

## Data Availability

No new data were created or analyzed in this study. Data sharing is not applicable to this article.
